# Amid COVID-19 pandemic, are non-COVID patients left in the lurch?

**DOI:** 10.12669/pjms.37.2.3536

**Published:** 2021

**Authors:** Laima Alam, Syed Kumail Hasan Kazmi, Mafaza Alam, Varqa Faraid

**Affiliations:** 1 Laima Alam, FCPS. Junior Consultant Gastroenterology, Bahria Town International Hospital, Phase VIII, Rawalpindi, Pakistan; 2 Syed Kumail Hasan Kazmi, FCPS. Fellow Gastroenterology, Pak Emirates Military Hospital, Rawalpindi, Pakistan; 3 Mafaza Alam, Registrar Operative Dentistry, Armed Forces Institute of Dentistry, Rawalpindi, Pakistan; 4 Varqa Faraid, General Dentist, Tooth Works Dental Clinic, Islamabad, Pakistan

**Keywords:** COVID-19, chronic disease, depression, health care, inequalities, pandemic

## Abstract

**Objectives:**

1) To explore the possible impact of the pandemic on the health seeking behavior of the patients, 2) To explore the relation of socio-demographics on the utility of health-care facilities.

**Methods:**

This cross-sectional study was conducted by enrolling all patients ≥15 years of age presenting to the Out-Patient-Department of three main public-hospitals after obtaining ethical committee approval. A questionnaire with validated Urdu translation was filled by each participant that included socio-demographic data, pre-Covid and Covid-19 era health seeking behaviors and the impact of the pandemic on the utilization of healthcare facilities. Data was analyzed using SPSS V.19.

**Results:**

A total of 393 patients were enrolled with a male preponderance (72%) and a median age range of 31-45 years. Fifty-eight percent of the study population was unemployed and 47.3% were seeking follow up care. The frequency of ER and multiple (>4 times) OPD visits were significantly decreased in the Covid-19 times whereas, the laboratory and radiology services were largely unaffected. A significant number of patients were not satisfied with the current healthcare facilities that was seen irrespective of the socio-demographic status. Emergency Room and radiology services were largely unaffected whereas, elective procedures and laboratory facilities were reported to be severely affected or delayed in relation to socio-demographic variables.

**Conclusions:**

Healthcare inequalities have widened and depression has shown a sharp rise during this pandemic. The over-burdened healthcare facilities at the verge of collapse may miss out on the chronic non-Covid patients which would ultimately lead to increased morbidity and mortality.

## INTRODUCTION

Due to the unprecedented conditions secondary to Covid-19 pandemic, the hospitals and emergency departments are overwhelmed with critically ill patients. The already fragile health care system of many developing as well as the developed countries has collapsed, making preventive techniques and effective infection control the major facets of clinical care.^1^ The importance of hospital readiness and establishment of fool proof triage systems for patient segregation and staff safety has been tremendously emphasized.^2^

Social distancing, frequent prolonged lockdowns, unemployment, lack of social support systems, fear of getting infected and overwhelmed hospitals have contributed adversely to the health seeking behavior of the community. An analysis from the United States showed a 40% reduction in STEMI presentation in Rhodes Island and a similar study reported a 50% reduction in emergency department visits (other than respiratory symptoms).^3^ This has led to the speculation that many patients with worrisome and serious symptoms might be avoiding the hospitals, causing delayed diagnosis and increased morbidity and mortality.^4^

Things might not be this simple for many low and middle income countries where the socio-demographic discrepancy has a significant effect on the availability of quality health care^5^ and the public, although willing to venture out of their homes, are denied health care due to affordability, accessibility or unavailability in the over-flowing community hospitals.^6^ For the similar reasons, chronic diseases that have been poorly managed in the socio-economically deprived community would face a further deterioration.

This is one of the few studies conducted at the community level to explore the possible impact of the pandemic on the health seeking behavior of patients and the delays the community has to suffer in the face of the over-burdened heath care facilities. Also, the discrepancy in availability of the health services faced in relation to socio-demographics was studied within the limitations.

## METHODS

This cross-sectional study was conducted by enrolling all patients, aged 15 years or more and freely consenting to participate in the study, visiting the OPDs of three large hospitals of the country namely Pak Emirates Military Hospital, Bahria Town International Hospital and Armed Forces Institute of Dentistry Rawalpindi after taking ethical committee approval from the concerned department (A/28/EC/196/2020 dated 01-07-2020). The patients were enrolled using convenience sampling and the sample size was calculated using OpenEpi sample size calculator with 5% margin of error, 95% confidence interval and frequency of outcome factor set at 50%.

The questionnaire was translated in to Urdu language and was back translated in to English by a translator completely blind to the study. This translation was then validated by two sources before putting to test.^7^ Data collection was started during the first week of July 2020 and the total duration of the study was one month.

### Statistical Analysis

To measure the internal consistency of the instrument, Cronbach’s alpha was calculated which produced a value of 0.78. Qualitative data was expressed as frequencies and percentages. Relation of health-seeking behavior with socio-demographic variables was seen using multinomial logistic regression. A value of <0.05 was considered statistically significant All analysis was done using SPSS V.19.

## RESULTS

A total of 393 patients, of age ≥15 years, attending the OPD were enrolled with male preponderance (72%) and the age range of 31-45 years representing the majority (38%). Fifty-eight percent (58%) of the study population was unemployed and majority hailed from urban residency (77.6%). Almost half of the study sample (47.3%) was seeking follow up care (Table-I).

**Table-I:** Demographics and consultation parameters of patients enrolled (n=393).

*Variables*	*Frequency (n%)*	*P value*
***Gender***		
Male	283(72)	<0.001
Female	110(28)	
***Age (years)***		
15-30	87(22)	<0.001
31-45	150(38)	
46-60	89(22.6)	
61-75	47(12)	
76-90	19(5)	
***Socioeconomic status***		
Low	111(28)	<0.001
Middle	255(65)	
High	27(7)	
***Residence***		
Urban	305(77.6)	<0.001
Rural	88(22.4)	
Education		
Illiterate	15(3.8)	<0.001
Read and write	17(4.3)	
Primary	23(6)	
High school	54(13.7)	
College	184(47)	
University	63(16)	
Post grad/ doctorate	37(9)	
***Family size***		
<5	182(46.3)	<0.001
6-7	146(37.2)	
≥8	65(16.5)	
***Employment status***		
Employed	24(6)	<0.001
Self employed	141(36)	
Unemployed	228(58)	
***Chronic illness***		
Yes	198(50.4)	<0.001
No	195(49.6)	
***Nature of consultation***		
Follow up	186(47.3)	<0.001
New appointment	207(52.7)	
Services availed	<0.001
Gastroenterology	52(13.2)	
General medicine	141(36)	
Pulmonology	49(12.5)	
G surgery	22(5.6)	
***Cardiology***	3(0.8)	
Neurology	3(0.8)	<0.001
Dental	88(22.4)	
Oncology	3(0.8)	
Psychiatry	8(2)	
Endocrinology	4(1)	
Nephrology	6(1.5)	
Dermatology	12(3.1)	
***Pre-Covid OPD visits over 3 months***		
0	132(33.6)	
1-2	102(26)	
3-4	51(13)	
>4	108(27.5)	
***Peri-Covid OPD visits over 3 months***		
0	88(22.4)	
1-2	270(69)	
3-4	28(7)	
>4	7(2)	<0.001
***Pre-Covid ER visits over 3 months***		
0	225(57.3)	
1-2	151(38.4)	
3-4	13(3.3)	
>4	4 (1)	
***Peri-Covid ER visits over 3 months***		
0	270(69)	
1-2	103(26)	
3-4	13(3.3)	
>4	7(2)	

The frequency of multiple (>4 times) OPD visits was significantly decreased in the Covid times either due to non-availability of services for follow up of chronic illnesses or deliberate avoidance whereas, the laboratory and radiology services were largely unaffected (Table-II). Patients admitted to deliberately missing their regular OPD and ER visits during the pandemic with longer than usual waiting time for all elective procedures, laboratory tests and visits (Table-III). Radiology services were reported to be prompt with no major delays.

**Table-II:** Health seeking behavior in peri-Covid era with refusal of services over a period of three months.

*Refusal of services over 3 months*	*0*	*1-2 times*	*3-4 times*	*>4 times*	*P value*
OPD	196(49.6)	83(21)	25(6.4)	89(23)	<0.001
ER	264(67)	112(28.5)	17(4.3)	0	<0.001
Lab	251(64)	45(11.5)	7(2)	90(23)	<0.001
Radiology	310(79)	57(14.5)	18(4.6)	8(2)	<0.001

**Table-III:** Evaluation of health care facilities in the Covid era using a five-point scale.

*Item*	*Strongly disagree*	*Disagree*	*Undecided*	*Agree*	*Strongly agree*	*p*
Do you deliberately avoid going to ER?	46(11.7)	98(25)	52(13)	140(35.6)	56(14)	<0.001
Do you deliberately avoid going to your regular OPD visits?	40(10)	134(34)	48(12)	121(31)	50(13)	<0.001
Do you have to wait longer than usual in ER?	53(13.5)	91(23)	69(17.6)	121(31)	59(15)	<0.001
Do you have to wait longer than usual in OPD?	54(13.7)	97(24.7)	57(14.5)	109(27.7)	75(19)	<0.001
Do you have to wait longer than usual for scheduled surgery/procedure?	52(13.2)	33(8.4)	100(25.4)	73(18.6)	135(34.4)	<0.001
Do you have to wait longer than usual at the lab?	44(11.2)	44(11.2)	75(19.1)	187(47.6)	43(11)	<0.001
Do you have to wait longer than usual at the radiology?	45(11.5)	112(28.5)	108(27.5)	98(25)	30(7.6)	<0.001
Do you have to wait longer than usual at the pharmacy?	43(11)	38(10)	63(16)	204(52)	45(11.5)	<0.001
Do you have to wait longer than usual for parking facility?	52(13.2)	131(33.3)	102(26)	85(21.6)	23(6)	<0.001
Do have to travel long distance to reach the heath-care facility?	47(12)	35(9)	90(23)	134(34)	87(22)	<0.001
Are you satisfied from the current heath care in pandemic?	111(28.2)	67(17)	45(11.5)	107(27.2)	63(16)	<0.001
Do you think the heath care needs improvement during pandemic?	8(2)	20(5.1)	51(13)	137(35)	177(45)	<0.001
Do you feel the need for non-COVID hospitals?	16(4)	23(6)	58(15)	138(35)	158(40)	<0.001
Do you feel tele-medicine is the way forward?	99(25.2)	68(17.3)	63(17.3)	96(24.4)	67(17)	0.003
Would you like a separate waiting area in ER /OPD / Lab after being screened for Covid-19 at the hospital entrance?	13(3.3)	19(5)	42(11)	140(35.6)	179(45.5)	<0.001
Do you feel depressed because of the current health care system?	44(11.2)	35(9)	44(11.2)	173(44)	97(24.7)	<0.001
Are these conditions financially taxing for you?	46(11.7)	36(9)	41(10.4)	180(46)	90(23)	<0.001

A significant number of patients were not satisfied with the current healthcare facilities and relayed that improvements are necessary. Also the need for non-Covid hospitals or at least a separate waiting area in the OPD, ER and laboratory after a thorough triage was strongly advocated. Majority of the patients were depressed and were facing financial constraints (Table-III). ER and radiology services were largely unaffected by socio-demographic variables whereas, the rest of the services were reported to be statistically affected by financial resources. (Table-IV). Patients with chronic illnesses requiring frequent hospital visits iterated repeated refusals and longer than usual wait with a high level of dissatisfaction towards health services as a whole.

**Table-IV:** Relation of health seeking behavior with socio-demographic variables using multinomial logistic regression.

*Health seeking behavior*	*Chronic illness*	*Socioeconomic status*	*Residence*	*Employment status*
Denied OPD services	<0.001	<0.001	0.40	<0.001
Denied ER services	0.69	0.33	0.63	0.17
Denied radiology services	0.03	0.39	0.03	0.09
Denied lab services	<0.001	<0.001	0.03	<0.001
Avoiding ER	0.02	0.59	0.68	<0.001
Avoiding OPD visits	<0.001	0.20	0.76	<0.001
Longer wait at ER	<0.001	0.06	0.22	<0.001
Longer wait at OPD	<0.001	0.03	0.86	0.005
Longer wait at pharmacy	<0.001	0.01	0.77	0.02
Longer wait at lab	<0.001	0.04	0.39	0.008
Longer wait at radiology	<0.001	0.20	0.30	<0.001
Longer wait for procedure	0.12	<0.001	0.48	0.004
Non-satisfaction from healthcare	0.02	<0.001	0.38	0.03

## DISCUSSIONS

Delay in seeking or providing medical attention may lead to morbidity and mortality more devastating than the Covid-19 pandemic itself.^8^ In multi-centre studies from Spain and Austria, 39.4 to 40% reductions in acute STEMI activations were reported during the months of March and April^9^. A decrease in stroke presentation was also seen in the United States and was speculated to be due to social distancing, decrease transportation, fear of getting infected and in some instances altruist approach to help the health care staff by not over-crowding the hospitals unnecessarily.^9^ In light of the current situation where most of the resources have been diverted to fight the pandemic, patients with multiple co-morbidities requiring frequent follow ups are the ones suffering the most.^10^

Since our study set-up was urban with two public and one welfare hospital, patients belonging to low and middle social classes were the main representatives. Unemployed patients were venturing out more to seek health care probably because of more time at hand, increased psychosomatic symptoms or fear of getting worse. The pandemic didn’t significantly affect the OPD visits as a whole, showing that the people were eager to get healthcare, although refusal of OPD services was statistically related to the social status of the population. ER and radiology services were largely unaffected by socio-demographics but the elective procedures and services were significantly affected or delayed with socially affluent population being able to get appointments in private set ups. The residence also did not affect the health seeking behavior and the presence of psychological distress significantly, as people could travel in and out of the cities following SOPs.

Socio-economically under-privileged people with larger families were avoiding OPD and ER visits probably due to overburdened set ups and refusal of services at public hospitals. The health-related inequalities, though prevalent in our community, have widened further and would tremendously affect the basic human right to quality care.^11^

The lack of satisfaction towards healthcare facilities was also seen to be related to the socio-demographic status in our study. There was a statistically significant relation between the presence of depression secondary to quality-of-health-care provision and social and employment status which was studied in a similar study showing a sharp rise in depression and stress scores for younger, unemployed population confined to homes.^12^ Also unemployed, low to middle class population reported greater financial strain in relation to increased travel expenditure for finding a hospital in our study.

These unprecedented conditions have transformed all the aspects of medical and surgical patient care. Telemedicine is emerging as a safe alternative for in-person consultations and a significant number of patients in our study showed their willingness towards telemedicine by sitting in the confines of their homes in contrast to a study that showed poor public acceptance.^13^ Telemedicine consultation with medical/surgical record review can help tremendously with segregating patients presenting with conditions manageable at home, prescription reviews and counseling versus those needing urgent medical attention.^14^

Also an effective system of triaging patients at ER or a designated area isolated from the rest of the hospital, into low and high risk patients for COVID-19 infection would minimize the number of trainees/consultants exposed.^15^ Majority of our patients showed their concern regarding safe waiting area allocation. Clinical algorithms in lieu with the region’s cultural and economic conditions and a multi-disciplinary approach towards policy making are also strongly advocated.^16^

To mitigate the impeding collapse of health care, mobile health care services, mobile pharmacies with prescription refills, preliminary triaging at the community dispensaries and small hospitals should be encouraged.^17^ Also reinstitution of vaccination programs, DOTS for TB and other community efforts to stop the spread of other communicable diseases like HIV, HBV, HCV, Malaria and Dengue should be emphasized. Registered pharmacists may be recruited to help with patient counseling, medication information, simple monitoring like blood pressure and blood glucose, monitoring for adverse reactions, and addressing polypharmacy.^6^ Focus should be placed at prioritizing chronic disease care in the deprived locations.

### Limitation of the study

The study set up comprised of public and welfare hospitals which might have led to sample bias in relation to socio-demographics. Also the Obstetric and Gynae clinic and specialized surgical units were not a part of the study due to logistics and allocation of different buildings to these services.

## CONCLUSION

While robust research is going on for Covid-19 management, literature lacks the effective ways to mitigate the impeding healthcare disaster for non-Covid emergency and non-emergency situations. Healthcare inequalities have widened leading to depression and financial taxation. The over-burdened health care facilities at the verge of collapse may miss out on the chronic non-Covid patients which would ultimately lead to increased morbidity and mortality.

### Authors’ Contribution:

**LA:** Contributed to the idea, design and drafting of the manuscript, data collection, statistical analysis and literature review.

**KHK:** Contributed to data collection.

**MA:** Contributed to data collection and literature review.

**VF:** Contributed to data collection.

All authors take equal responsibility for the accuracy and integrity of the work.
